# Investigation of the Physiological and Antioxidant Properties of the Medicinal and Endemic *Hypericum bilgehan-bilgilii* Species Under Different Cultivation Methods

**DOI:** 10.3390/biology14091302

**Published:** 2025-09-20

**Authors:** Huseyin Turker

**Affiliations:** Biotechnology Department, Faculty of Sciences, Niğde Ömer Halisdemir University, 51240 Nigde, Türkiye; huseyinturker@ohu.edu.tr

**Keywords:** antioxidant activity, *Hypericum bilgehan bilgilii*, local endemic, medicinal plant, physiological properties, potential phytochemicals

## Abstract

*Hypericum bilgehan bilgilii* is a locally endemic species belonging to the genus *Hypericum*. Considering that the related plant has pharmacological and medicinal potential like other *Hypericum* species, this study has been conducted to determine its physiological and antioxidant activities. Plant extracts obtained through three different cultivation methods (wild, cultivated in a laboratory environment in peat, and cultivated using tissue culture methods) were used to determine physiological, biochemical, and antioxidant activity analyses. (The abbreviations for the cultivation methods are indicated as, respectively, W, in vivo in lab, in vitro in lab). In this study, the highest photosynthetic pigment content, total protein content, proline content, malondialdehyde (MDA) content, phenylalanine ammonium lyase (PAL) activity, antioxidant activity (2,2-Diphenyl-1-picryl hydrazyl radical scavenging activity, total phenolic content, carotenoid) values were determined for the first time in the in vitro in lab group in *H. bilgehan bilgilii* plants grown under three different cultivation methods. Superoxide dismutase (SOD) and catalase (CAT) enzyme activities were observed at the highest levels in the wild group. Based on these parameters, the physiological, biochemical, and antioxidant activities of the *H. bilgehan bilgilii* plant were studied for the first time under different cultivation conditions, producing new results. According to these results, it was determined that in vitro cultivated plants have higher physiological parameters and antioxidant content. This study has also demonstrated the significance of tissue culture, a biotechnological method, in enhancing bioactive compounds, and that in vitro cultures could be used as an alternative to traditional methods for producing larger quantities of bioactive compounds. In addition, establishing in vitro cultures for endemic, endangered, and medically significant species will enable the sustainable use of the local endemic species *H. bilgehan-bilgilii*, facilitate large-scale production of phytochemicals, and provide a valuable plant resource and information for future comprehensive phytochemical studies.

## Simple Summary

In the present study, various physiological parameters, antioxidant levels, and antioxidant activities have been determined in leaf extracts of the endemic *Hypericum bilgehan-bilgilii* species, which has potential as a medicinal plant, obtained using three different cultivation methods. When evaluated in terms of antioxidant capacity, it has been found that plants grown in in vitro cultures possessed higher antioxidant properties. This study has yielded important results for the discovery of valuable phytochemicals offered by nature’s pharmacy.

## 1. Introduction

The genus *Hypericum* L. of the family Hypericaceae shows diversity in terms of morphological structure and constitutes the largest genus in terms of species [1,2,3]. There are about 500 species distributed almost all over the world [1,2,4,5,6]. The genus *Hypericum* contains approximately 110 taxa in Türkiye. Approximately 48% of the species distributed in Türkiye are in the country-specific endemic category [7]. Plant species belonging to the genus *Hypericum* are usually in the form of shrub(s) or herb(s) and rarely in the form of tree(s) [1,7]. The flowers are usually yellow or golden yellow. Several species of the genus *Hypericum* are cultivated worldwide as ornamental plants. However, the majority of the species belonging to the genus have a high medicinal value [3,8,9,10,11].

It has been reported that phenolic compounds and phenolic compound-derived secondary metabolites are the most abundant active ingredients in *Hypericum* species [12]. Due to the valuable secondary metabolites found in *Hypericum* species, it has been reported to show vital biological activities such as antioxidant, anti-inflammatory, antitumor, antihyperglycemic, antidepressant, hepatoprotective, and antimicrobial [8,13,14,15]. Phenolic compounds are thought to reduce the risk of many diseases, including cardiovascular and different types of cancer, due to their high antioxidant activity [13,16].

*Hypericum bilgehan bilgilii* is a locally endemic species and is distributed in 2000–2800 m altitude areas within the borders of Dumanlı Village in the Beyşehir district of Konya province in Türkiye. Since it survives in extreme conditions, it is thought to have high phenolic compounds and antioxidant levels, as in other *Hypericum* species. Although many studies have been carried out on the physiological, biochemical, and antioxidant properties of different *Hypericum* species in the literature [9,17,18,19,20,21,22], there are no studies on the physiological and antioxidant properties of *H. bilgehan bilgilii*. *H. bilgehan-bilgilii* lives in extreme conditions in its natural habitat, so it is exposed to various biotic and abiotic stress factors such as extreme heat, extreme cold, strong winds, water scarcity, grazing activities, small predator herbivores, and other human activities. It is thought that it survives by activating different defense mechanisms (such as phytochemicals unique to itself, antioxidant defense systems, and species-specific essential compounds) against these stresses. Therefore, this study has been carried out based on the assumption that the plant species in question contains different or more phytochemicals.

The paucity of research on the physiological and antioxidant properties of this species is a significant shortcoming, notably given its potential pharmacological value. Therefore, this study examined the antioxidant and physiological properties of the plant using leaf extracts obtained through different cultivation methods. In this study, the physiological, biochemical, and antioxidant properties of plants (leaves) obtained from three different cultivation methods (wild, cultivated in peat in a laboratory environment, and cultivated using tissue culture) of *H. bilgehan bilgilii* have been determined for the first time. (The abbreviations for the cultivation methods are indicated as, respectively, W, in vivo in lab, in vitro in lab).

## 2. Materials and Methods

### 2.1. Obtaining Plant Material

**Figure 1:**
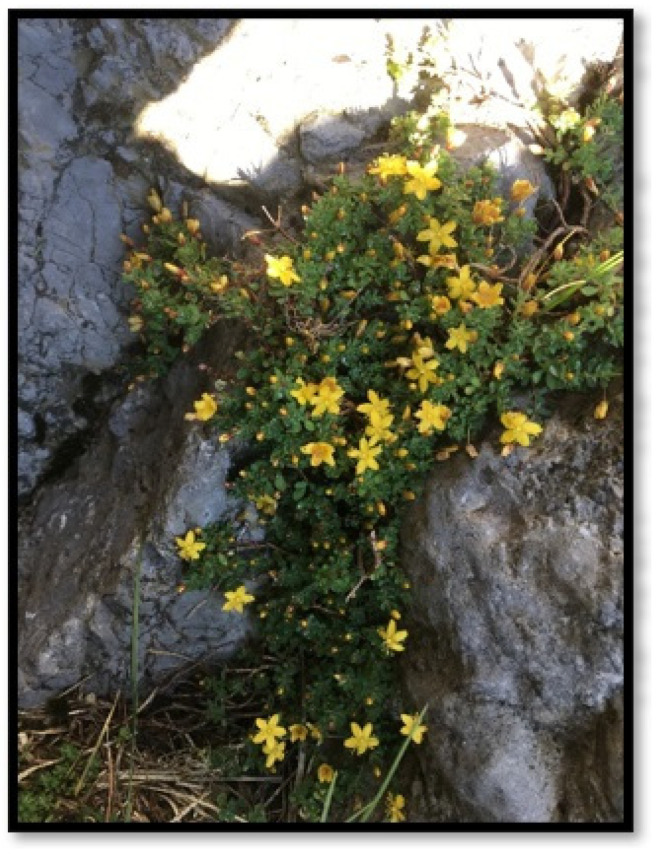
Local endemic in its natural range, *H. bilgehan-bilgilii*.

*H. bilgehan bilgilii* was collected from the wild-growing related plant Beyşehir/Konya/Türkiye (N37°29′21.66″–E31°19′35.81″). The species was identified by one of the authors, Dr. Ahmet Savran, from various flora or vegetation sources and deposited in the Herbarium of Niğde Ömer Halisdemir University (voucher number: Başköse and Savran 4600). *H. bilgehan bilgilii* (Figure 1) seeds were hand-collected in minimum quantities from 10 randomly selected plants and stored at 4 ± 2 °C in pots containing peat and in small, sealed plastic containers for in vitro cultures.

#### 2.1.1. Wild Plants

For the plant material, the required amounts of leaves of the plants provided were taken and used for wild plant analysis. The collected plant samples were transported to the laboratory using a cold chain.

#### 2.1.2. Grown Plants in Peat in the Laboratory Environment (In Vivo in Lab)

**Figure 2:**
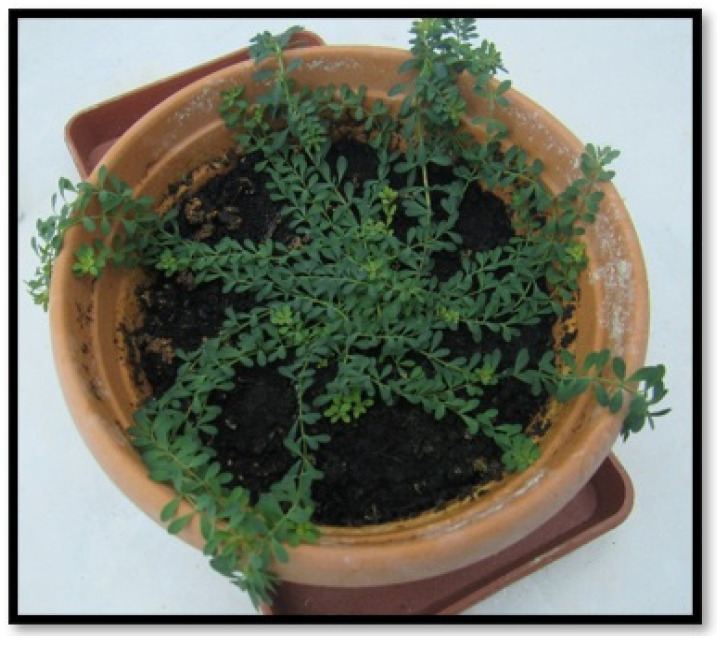
*H. bilgehan-bilgilii* grown in peat in the laboratory.

*H. bilgehan bilgilii* seeds were germinated in pots containing peat. The seeds were kept in 2.0 mg·L^−1^ gibberellic acid (GA) for 24 hours before being sown in the pots. This ratio was preferred since 2.0 mg·L^−1^ GA increases the germination percentage and duration of *H. bilgehan bilgilii* [5]. Seeds were planted in 10 pots with 5 seeds in each pot (since *H. bilgehan bilgilii* is a locally endemic and critically endangered species, a minimum of seeds was used). Seedling cultivation from seeds in pots (Figure 2) was carried out in a plant growth room with a temperature of 25 ± 2 °C, a photoperiod of 16/8 (light/dark), a constant humidity of 50 ± 5%, and a light intensity of 27 μmol m^−2^ s^−1^.

#### 2.1.3. In Vitro Culture Formation 

**Figure 3:**
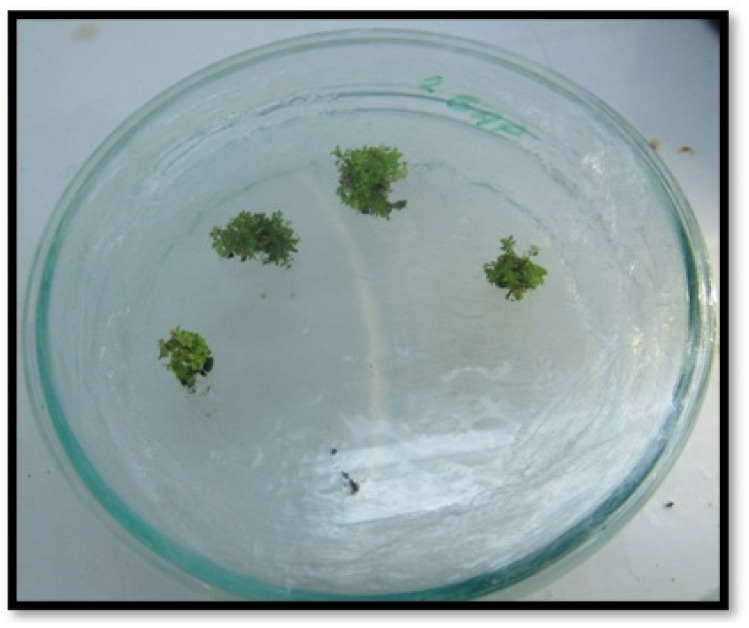
*H. bilgehan-bilgilii* micro seedlings growing on the MS medium containing 2.0 mg·L^−1^ BAP.

*H. bilgehan-bilgilii* seeds were germinated on an MS medium containing 2.0 mg·L^−1^ GA [23], and nodal explants from germinated plants have been used for callus and shoot formation. It has been found in previous studies that benzyl amino purine (BAP) and 2,4-dichlorophenoxy acetic acid (2,4-D) are the best plant growth regulators for callus formation in *H. bilgehan-bilgilii* [6]. Therefore, MS media containing 0.8 mg·L^−1^ BAP and 0.4 mg·L^−1^ 2,4-D were used for callus formation. Callus and shoot formation studies have been carried out in Petri dishes (containing MS medium). A total of 3% sucrose and 4.405 g L^−1^ MS was used. The pH of the MS media used in the study was adjusted to 5.8 and sterilized in an autoclave at 121 °C for 25 min. In previous studies, it was determined that the hormone and concentration of BAP and 2.0 mg·L^−1^ is the best promoter of shoot growth for *H. bilgehan-bilgilii*. Consequently, the calluses were subcultured on the MS medium containing 2.0 mg·L^−1^ BAP for shoot development (Figure 3), and many seedlings were formed. The leaves of these seedlings were utilized for physiological and biochemical analyses. All applications in the study were performed in three repetitions.

### 2.2. Conducted Physiological Analyses

#### 2.2.1. Photosynthetic Pigment Content (PPC)

The method given by Witham et al. [24] for determining the chlorophyll content was used. The absorbance levels of acetone extracts of leaf samples taken randomly from the treatment groups was measured at 645 nm and 663 nm in a Boeco spectrophotometer. The amounts of Chlorophyll a (Chla), Chlorophyll b (Chlb), and total Chlorophyll (total Chl) were calculated and expressed as mg.g^−1^ fresh leaf tissue. All measurements made in the study were carried out in three repetitions.

#### 2.2.2. Total Protein (TP) Quantities

To investigate the extent to which different cultivation methods affect protein content in plants, total protein analysis was conducted. A total of 1 g of each fresh leaf sample has been extracted in Na-phosphate buffer, pH 7.8, and 0.05 M, using an ice bath. The extract(s) have been centrifuged at 13,000 rpm for 30 min at +4 °C. TP has been determined according to the method of Bradford [25] using Bovine Serum Albumin (BSA) standards. TP content has been calculated as mg·g^−1^ fresh weight. All measurements made in the study were carried out in three repetitions.

#### 2.2.3. Proline Amounts (PA)

To determine the extent to which different cultivation methods can cause oxidative stress or damage in plants, proline analysis was performed. Proline amounts have been determined according to the modified method of Bates et al. [26]. Leaf sample(s) were in 3% (*w*/*v*) sulfosalicylic acid solution homogenized and centrifuged. The supernatant was transferred into a tube to which glacial acetic acid and acid ninhydrin solution was admixed. The tubes were in a boiling water bath, incubated for 1 h, and then cooled to room temperature. After adding toluene, the mixture was stirred with a vortex device, and the toluene and aqueous phases were allowed to separate. The absorptivity of the toluene phase was detected at 520 nm in a spectrophotometer. The concentration was calculated from the proline standard curve and expressed as μmol/g FW. All measurements made in the study were carried out in three repetitions.

#### 2.2.4. Malondialdehyde Content (MDA)

To determine the extent to which different cultivation methods can cause stress or damage to plants, the amount of lipid peroxidation was estimated by measuring MDA content. MDA contents were determined according to the modified method of Zhou and Leul [27]. A total of 0.2 g of fresh plant samples were homogenized with 4 mL of 0.1% trichloroacetic acid (TCA) solution and then centrifuged at 12,500 rpm for 5 min. A total of 1 mL of the supernatant was transferred to unused tubes, and 4 mL of 0.5% thiobarbituric acid (TBA) containing 20% TCA was added. The samples were kept in a 95 °C water bath for 30 min, and then the samples were quickly cooled and centrifuged at 10,000 rpm for 10 min. After centrifugation, the absorbance readings of the samples were taken at 532 and 600 nm wavelengths using a spectrophotometer. All measurements made in the study have been carried out in three repetitions.

#### 2.2.5. Total Phenolic Content (TPC)

TPC has been determined according to the modified method of Singleton and Rossi [28]. A total of 0.1 g leaf samples were homogenized with 5 mL of 80% methyl alcohol and then incubated at 80 °C for 15 min. After incubation, centrifugation was performed at 10,000 rpm for 10 min. The Folin–Ciocalteu method was used to determine the total phenolic content [28]. Total of 100 μL of the extract was incubated with 10 times diluted Folin–Ciocalteu for 5 min at room temperature, and then 750 μL of sodium bicarbonate solution was added to the present solution and incubated again for 90 min at room temperature in the dark. The absorbance values were measured at 765 nm in a spectrophotometer, and the phenolic content was calculated using a gallic acid standard. All measurements made in the study were carried out in three repetitions.

#### 2.2.6. Determination of Phenylalanine Ammonium Lyase (PAL) Activity

A total of 0.1 g of each sample was homogenized with 50 mM sodium phosphate buffer, pH 6.5, containing 1% polyvinylprolidone (PVP) and 1 mM Phenylmethylsulfonyl (PMSF). The homogenate was then centrifuged at 10,000 rpm for 25 min at 4 °C. The supernatant was then taken and used for the determination of PAL activity. The method of Pascholati, et al. [29] was used to determine PAL activity. A total of 100 μL of enzyme extract and 1000 μL of 0.2% phenylalanine solution was incubated at 37 °C, and the conversion of L-L-phenylalanine to trans cinnamic acid was measured at 290 nm absorbance (Thermo Scientific UV Spectrophotometer, Waltham, MA USA, Thermo Fisher Scientific). PAL activity was determined by preparing a standard of cinnamic acid and expressed as μmol cinnamic acid h^−1^.

### 2.3. Identification of Antioxidant Quantities and Activity

In order to determine whether different cultivation methods affect the amount and activity of antioxidants in plants, antioxidant content and activity analyses have been performed.

#### 2.3.1. Superoxide Dismutase (SOD) Enzyme Activity

SOD enzyme activity was measured according to the method of Beauchamp and Fridovich [30]. This method is based on “inhibition of the photochemical reduction of nitroblue tetrazolium (NBT) at 560 nm by the SOD enzyme in the sample”. After adding 3 mL of reaction mixture containing 50 mM sodium phosphate buffer, 33 μM NBT, 0.66 mM EDTA, 10 mM L^−1^ Methionine (Me), and 0.0033 mM Riboflavin (Rf) to the supernatants at 300 μmol^−1^ m^−1^ s^−1^ light intensity and room temperature, the reaction was allowed to take place for 10 min. The absorptance levels of the samples were obtained at 560 nm using a Thermo Scientific UV Spectrophotometer. Enzyme activity was defined as EU mg protein^−1^.g fresh weight ^−1^, calculated as the amount of SOD required for 50% inhibition of NBT as 1 enzyme unit.

#### 2.3.2. Catalase (CAT) Enzyme Activity

The activity assay of the CAT enzyme was performed according to Bergmeyer [31]. According to this method, 0.05 M Na-phosphate buffer (pH 7.0), 3% H_2_O_2_, and 1 mM EDTA was added to the supernatants, and the absorption change due to H_2_O_2_ consumption has been observed in a Thermo Scientific UV Spectrophotometer at 240 nm wavelength for 1 min. The amount of μmol H_2_O_2_ consumed per minute has been determined as 1 enzyme unit, and the specific enzyme activity at 240 nm was expressed as enzyme units mg protein. g fresh weight^−1^.

#### 2.3.3. 2,2-Diphenyl-1-Picryl Hydrazyl (DPPH) Radical Scavenging Activity

DPPH radical scavenging activity was determined according to the method of Brand-Williams, et al. [32]. To determine the antioxidant activity of *H. bilhehan-bilgilii* extracts, a series of dilutions was prepared using 0.1 mM DPPH solution, the respective plant extract(s), and methanol. A total of 100 μL of each dilution was added to 2.9 mL of DPPH solution and incubated at room temperature for 15 min. After incubation, the absorbance values of the samples were measured at 517 nm. Radical scavenging activity has been calculated according to the formula (% DPPH AB-AS/AB × 100).

AB: Blank absorbance value

AS: Sample absorbance value

#### 2.3.4. Determination of Carotenoid Amount

The absorbance value measured at 450 nm wavelength of the extracts prepared according to the method of [24] has been placed in the equation, and mg carotenoid content in 1 g of leaf fresh weight has been calculated.

### 2.4. Statistical Analysis (SA)

The data of all measurements and analyses was assessed with the Tukey testTukey [33] with a significance level of *p* < 0.05 using the Analysis of Variance (Multiple Range Test) in SPSS version 16.0 program. The standard error (SE) and standard deviation (SD) of the means was also determined in the same program.

## 3. Results

Since global climate change affects endemic, local endemic, endangered, and endemic plants with valuable bioactive compounds more, in vitro cultures of endemic plants with economic importance and potential to form a beneficial herbal medicine source should be established, and various physiological and biochemical analyses should be carried out to better understand the mechanisms involved. This study has determined some physiological and antioxidant properties in *H. bilgehan-bilgilii*, an endemic species with medicinal potential, grown under three different cultivated conditions.

### 3.1. Quantification of Photosynthetic Pigment Content

**Figure 4:**
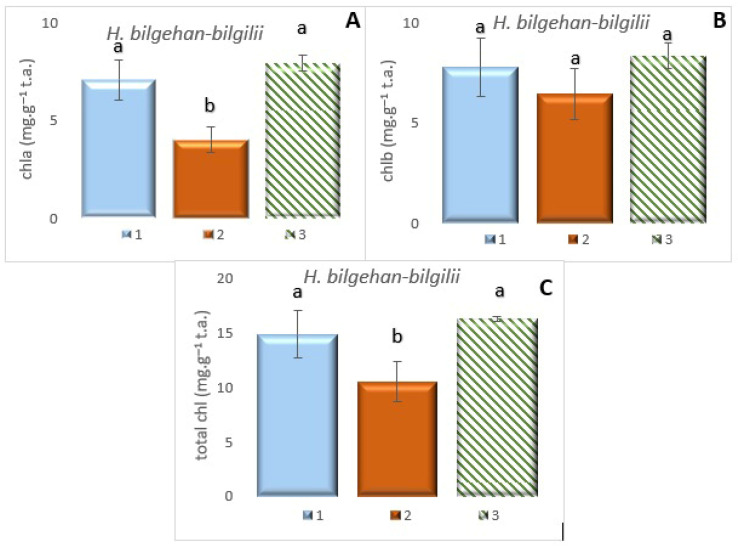
Chl a (**A**), chl b (**B**), and t chl (**C**) in the leaves in different growing methods. 1: W, 2: in vivo in lab, 3: in vitro in lab. a is significantly (*p* < 0.05) different from b.

In the present study, the amounts of photosynthetic pigments have been determined as chlorophyll a (chl a), chlorophyll b (chl b), and total chlorophyll (t chl) in *H. bilgehan-bilgilii* grown in three different growing methods (Figure 4).

It has been determined that the highest content of chl a, chl b, and t chl amounts in all study groups has been in the “in vitro in lab” group. The lowest photosynthetic pigment content has been observed in the “in vivo in lab” among all groups. It has been found that the percentage increase between the lowest and the highest content in chl a, chl b, and t chl amounts has been 96.02%, 30.10%, and 55.50%, respectively (*p* < 0.05).

It is known that the growth, development, bioactive substance production, and yield of plants are directly related to photosynthesis. In this study, it has been revealed that plants cultivated by the in vitro culture method have a higher amount of photosynthetic pigment content.

### 3.2. Identification of Total Protein Quantities 

**Figure 5:**
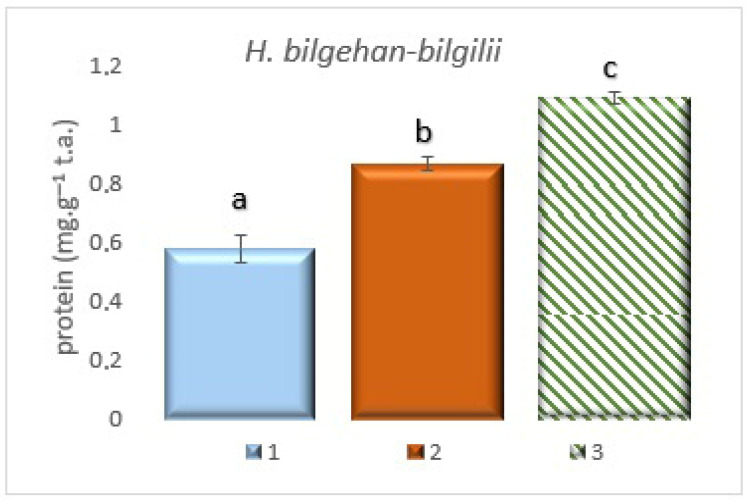
The total protein content in different growing methods. 1: W, 2: in vivo in lab., 3: in vitro in lab. a, b, and c are significantly (*p* < 0.05) different from each other.

TP amounts were detected in the *H. bilgehan-bilgilii* plant species grown in different growing methods. The highest TP amount has been determined in the treatment group in vitro in lab. The least amount of TP has been found in the “W” group (*p* < 0.05). The increase in TP amount in the group “in vitro in lab” is 89.4% compared to the “W” group (Figure 5). Although protein levels are lower in plants living in their natural habitat, higher SOD and CAT activities have been observed. It is thought that these plants stimulate enzymatic antioxidant pathways against oxidative stress, thereby defending themselves against various biotic and abiotic stresses. The determined increases are at the level of significance.

### 3.3. Proline Amount Determination

**Figure 6:**
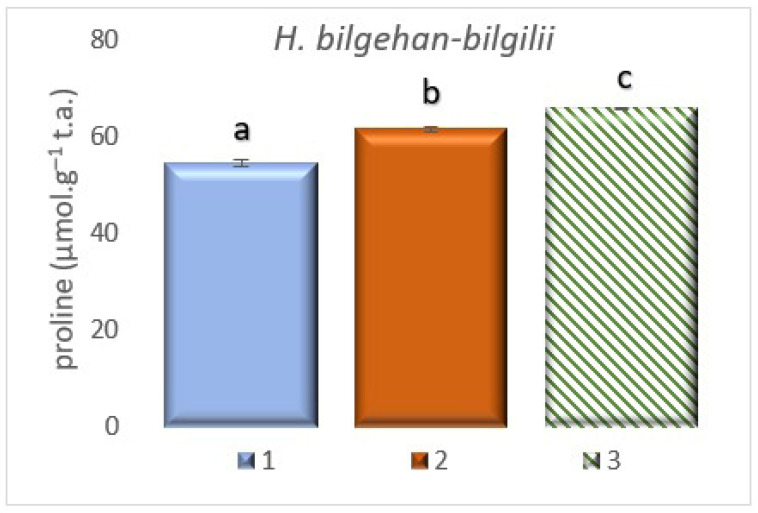
Proline amounts in different growing methods. 1: W, 2: in vivo in lab, 3: in vitro in lab. a, b, and c are significantly (*p* < 0.05) different from each other.

The study found proline amounts for the *H. bilgehan-bilgilii* species, and the highest proline amount has been determined in the “in vitro in lab” group. The lowest amount of proline has been detected in the “W” group (*p* < 0.05) (Figure 6). It has been observed that the group “in vitro in lab” contained 20.61% more proline than the “W” group. The determined increases are at the level of significance.

### 3.4. Detection of Malondialdehyde (MDA) Contents

**Figure 7:**
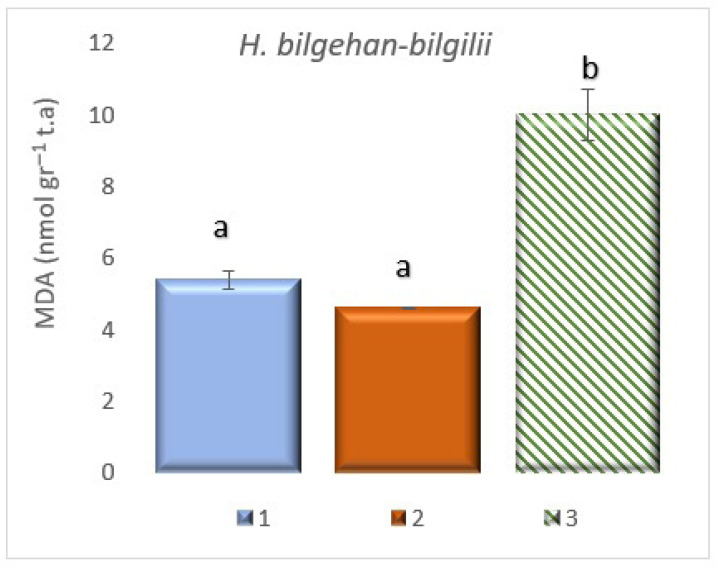
MDA content in different growing methods. 1: W, 2: in vivo in lab, 3: in vitro in lab. a is significantly (*p* < 0.05) different from b.

The highest level of MDA content in *H. bilgehan-bilgilii* grown in different growth methods has been found in the group “in vitro in lab”. The group with the lowest MDA content has been determined as the group “in vivo in lab” (*p* < 0.05). It has been observed that the MDA content increased approximately two times in the group “in vitro in lab” compared to the group “in vivo in lab” (Figure 7).

### 3.5. Quantification of Total Phenolic Content 

**Figure 8:**
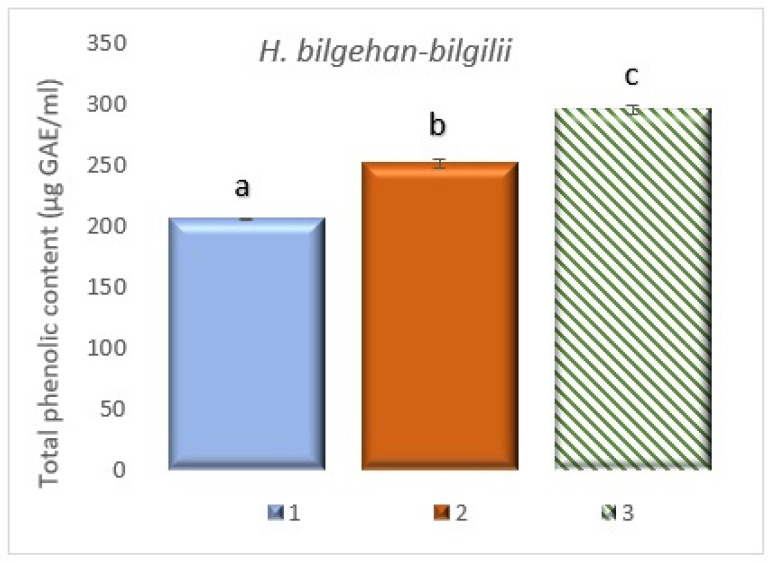
Total phenolic content in different growing methods. 1: W, 2: in vivo in lab, 3: in vitro in lab. a, b, and c are significantly (*p* < 0.05) different from each other.

The TPC of *H. bilgehan-bilgilii* has been measured by using the Folin–Ciocalteu method, and it has been determined that the highest TPC is in the group “in vitro in lab”. The group “W” has been determined to have the lowest TPC. Group “in vitro in lab” has been found to have 43.74% more TPC than group “W” (Figure 8). The determined increases are at the level of significance. Phenolic compounds are among non-enzymatic antioxidants and are known to be used in reducing the risk of, preventing, or treating many diseases, including cardiovascular diseases and various types of cancer, due to their high antioxidant activity [16,17,18,19]. It has been stated that phenolic compounds or phenolic compound-derived phytochemicals are obtained in greater quantities in in vitro cultures [34].

### 3.6. The Determination of Phenylalanine Ammonia Lyase (PAL) Activity 

**Figure 9:**
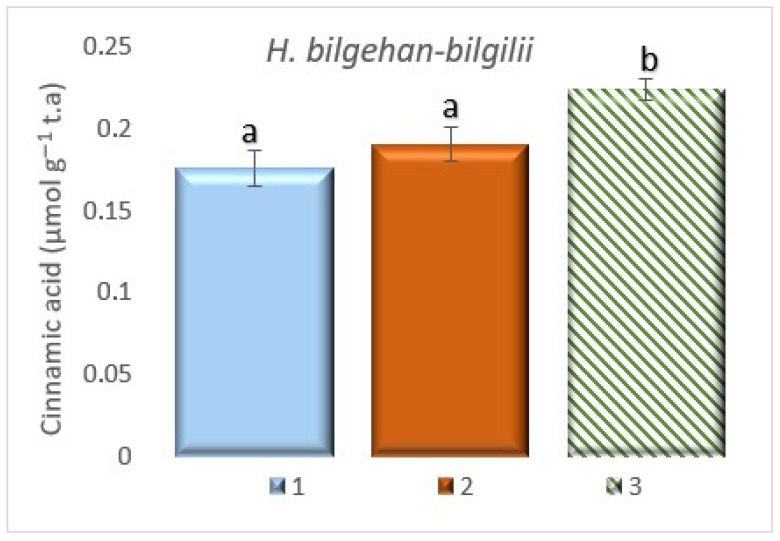
PAL activity in different growing methods. 1: W, 2: in vivo in lab, 3: in vitro in lab. a is significantly (*p* < 0.05) different from b.

In the PAL activity study for *the H. bilgehan-bilgilii* species grown in different conditions, the highest PAL activity has been observed in the group “in vitro in lab”. In the “W” group, the lowest PAL activity has been detected (*p* < 0.05). It has been determined that the “in vitro in lab” group has 12.66% more PAL activity than the “W” group (Figure 9).

### 3.7. Antioxidant Content and Activity Determination 

In the literature, several studies have been carried out on determining the antioxidant activity of plant extracts of different species, since other species belonging to the genus *Hypericum* contain high amounts of compound(s) capable of scavenging various types of reactive oxygen species (ROS). Due to the high antioxidant properties of *Hypericum* species, their use for different pathologies related to oxidative stress, such as eczema, skin wounds, and inflammation, has been expressed. However, it is thought that antioxidant properties may be higher and/or differ in different *Hypericum* species or plants living in extreme conditions.

#### 3.7.1. The Detection of Superoxide Dismutase (SOD) Enzyme Activity 

**Figure 10:**
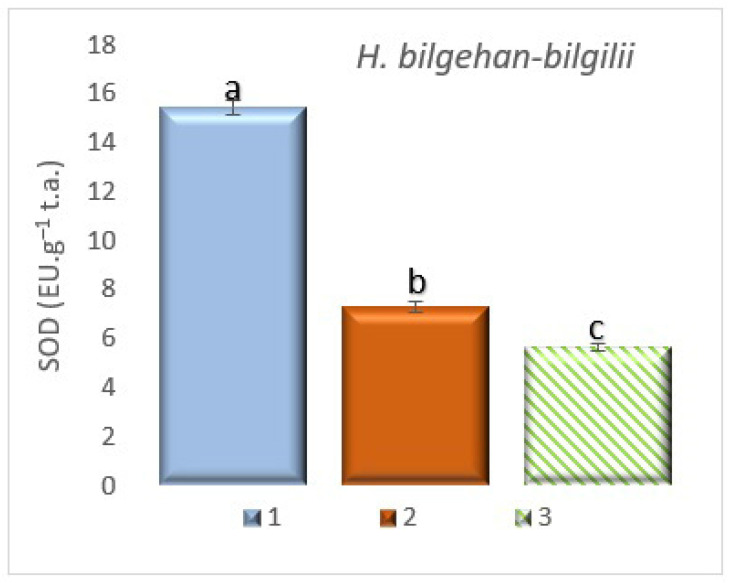
SOD activity in different growing methods. 1: W, 2: in vivo in lab., 3: in vitro in lab. a, b, and c are significantly (*p* < 0.05) different from each other.

In the analysis of the determination of SOD enzyme activity, it has been determined that the highest SOD enzyme activity is in the “W” group and the lowest SOD enzyme activity is in the “in vitro in lab” group. It has been found that the highest SOD enzyme activity was 63.3% higher than the lowest SOD enzyme activity (Figure 10). The determined increases are at the level of significance. SOD is among the enzymatic antioxidants. It is thought that the phenylpropanoid pathway is stimulated in plants obtained by in vitro culture methods. The higher PAL and TPC values in in vitro culture confirm this situation. Considering the results of SOD and CAT enzyme activities together, it is thought that the plant material used in the study activated the enzymatic antioxidant pathway mechanisms because it was exposed to more biotic stress factors in the W group’s natural habitat.

#### 3.7.2. The Determination of Catalase (CAT) Enzyme Activity 

**Figure 11:**
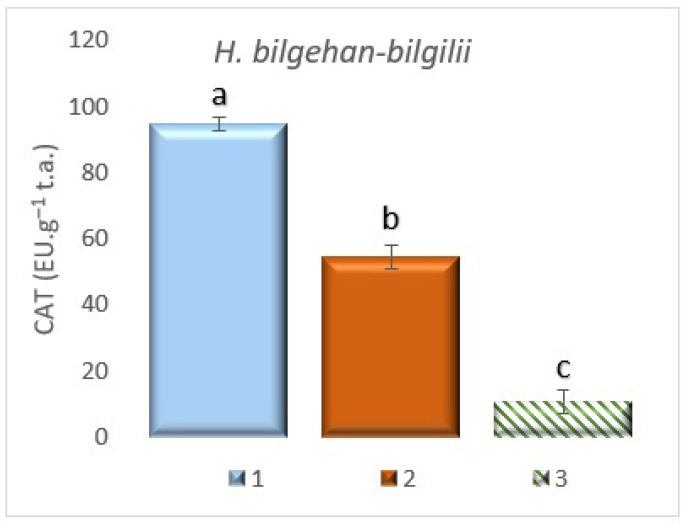
CAT enzyme activity in different growing methods. 1: W, 2: in vivo in lab., 3: in vitro in lab. a, b, and c are significantly (*p* < 0.05) different from each other.

CAT enzyme activity has been analyzed in *H. bilgehan-bilgilii* plants grown under different growing methods, and it has been determined that the highest CAT enzyme activity is in the “W” group. The lowest CAT enzyme activity has been found in the “in vitro in lab” group (*p* < 0.05). It has been observed that the “W” group had 88.6% more CAT enzyme activity than the “in vitro in lab” group (Figure 11). These findings suggest that the reason enzymatic antioxidant activities such as SOD and CAT are low in plants grown in vitro is thought to be due to the promotion of other non-enzymatic antioxidant defense system pathways, such as phenolic compounds and carotenoids in the plant. The fact that the most abundant and highly antioxidant compounds in *Hypericum* species are of phenolic compound origin supports this idea.

#### 3.7.3. Quantification of 2,2-Diphenyl-1-picrylhydrazyl (DPPH) Radical Scavenging Activity

**Figure 12:**
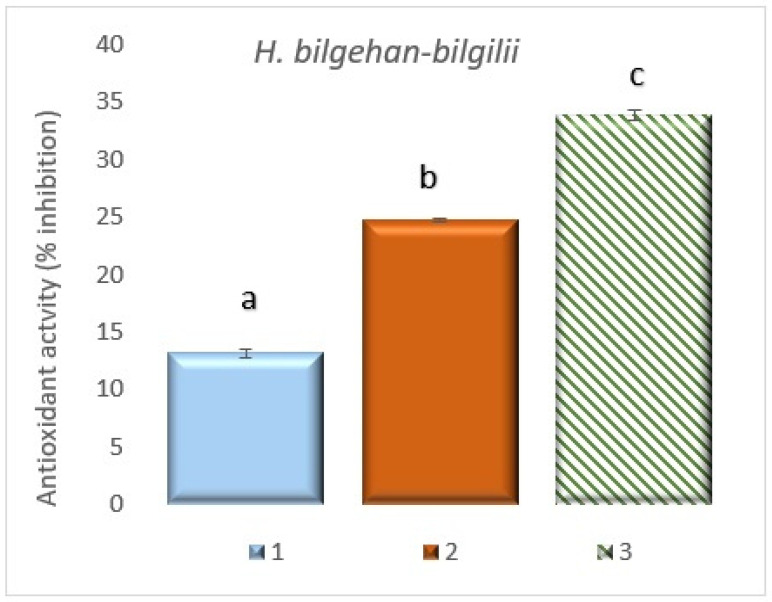
DPPH radical scavenging activity in different growing methods. 1: W, 2: in vivo in lab, 3: in vitro in lab. a, b, and c are significantly (*p* < 0.05) different from each other.

DPPH radical scavenging activity assay has been performed to determine the total antioxidant activity of the *H. bilgehan-bilgilii* plant. As a result of the analysis, the highest radical scavenging activity has been determined in the “in vitro in lab” group. The lowest radical scavenging activity has been detected in the “W” group. It has been concluded that the “in vitro in lab” group with the highest radical scavenging activity has 2.56 times more antioxidant activity than the “W” group with the lowest radical scavenging activity (Figure 12). The determined increases are at the level of significance. These results demonstrate that biotechnological methods are quite effective in increasing antioxidant levels. It is known that phytochemicals with high antioxidant effects, such as phenolic compounds, carotenoids, and phenylpropanoids, are important for pharmacology, medicine, and the food industry, and it has been determined that they can be obtained in greater quantities through in vitro cultures.

#### 3.7.4. Determining the Amount of Carotenoids 

**Figure 13:**
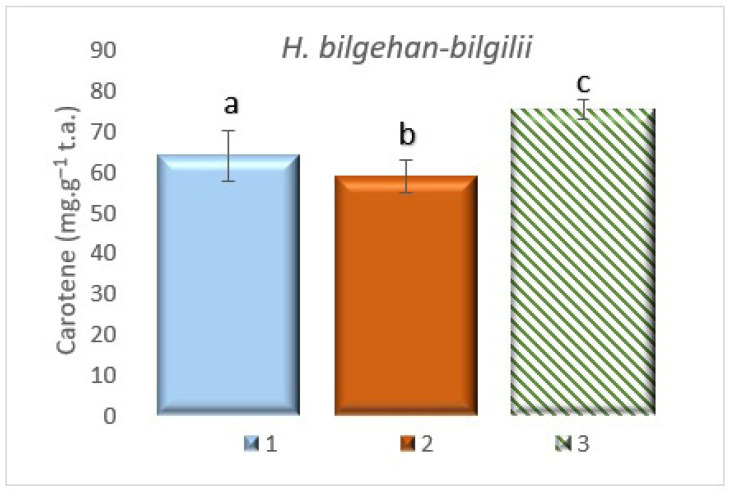
Carotenoid amounts in different growing methods. 1: W, 2: in vivo in lab, 3: in vitro in lab. a, b, and c are significantly (*p* < 0.05) different from each other.

Carotenoid analysis, one of the non-enzymatic compounds with antioxidant properties, has been performed for *H. bilgehan-bilgilii* plants grown in different conditions. In the analysis, the highest carotenoid amount has been determined in the “in vitro in lab” group, and the lowest carotenoid amount has been determined in the “in vivo in lab” group (*p* < 0.05). It has been detected that the “in vitro in lab” treatment group, which has the highest amount of carotenoids, has 20.1% more carotenoids than the “in vivo in lab” group, which has the lowest amount of carotenoids (Figure 13). This study also showed that plants grown with tissue culture techniques, which is one of the biotechnological methods, contain higher amounts of antioxidant substance(s). Carotenoids play a crucial role in the continuation of plant progeny. Together with phenolic compounds, they are responsible for the brilliant colors. These valuable phytochemicals, offered by nature’s pharmacy, have been shown to act as antioxidants that protect membranes from damage caused by free radicals and delay aging processes [35]. The study found that plants obtained through in vitro culture contain higher amounts of carotenoids, which are considered essential for health.

## 4. Discussion

The study contributed to the sustainable utilization of this endemic plant by investigating the physiological and antioxidant effects of different growing methods on the medicinal potential and local endemic *H. bilghan-bilgilii*. The other reason for the physiological, biochemical, and antioxidant activity analyses has been performed to create a study, as it is thought that this plant may have potential for future use in medicine and various fields.

Chlorophyll values (chl a, chl b, and t chl) have been correlated with each other in all treatment groups. It has been reported in previous studies that chlorophyll a and total chlorophyll values have positive or negative correlations with many parameters measured in plants [36]. It has been reported that the amounts of chlorophyll a and total chlorophyll in plants play an important role in the synthesis or formation rate of many bioactive substances [36,37,38]. Similar results have been observed in this study, and it has been determined that the findings are statistically significant. It is also thought that various physiological, biochemical, and antioxidant parameters are related to chlorophyll content. It has been reported that chlorophyll a and b amounts in *Hypericum* species and other plant species and the changes between them show the variability of the photosynthesis mechanism according to the environmental conditions in which the plant grows and that these changes affect the production pathways of secondary metabolite(s) that involve various physiological, biochemical, and antioxidant processes of the plant [36,37,39,40,41,42]. Furthermore, chlorophyll levels in plants can serve as indicators of growth, development, yield, stress status, and bioactive compounds, making chlorophyll analysis a fundamental tool. Therefore, determining chlorophyll levels is crucial because the regulation of mechanisms between energy systems and pathways in plants is also linked to the photosynthesis mechanism.

It has been stated that total protein amounts vary depending on altitude, environmental factors, and plant growing conditions. It has been reported that the change(s) observed have generally been observed as an increase in protein amounts. It has been explained that this may be related to reductase-type enzymes and that protein accumulation increases due to environmental factors. The increase in the amount of total phenols and other non-enzymatic antioxidants supports this situation [43]. In a study on *H. perforatum* L., it has been reported that changes in environmental factors increase the total protein content [44]. It has been stated that changes in the growth conditions of the plant, such as altitude, longitude, and latitude differences, are important factors in the production of bioactive substances [40]. In other studies, changes in total protein amounts have been determined in plant extracts obtained under different growing conditions [43]. Similar results have been obtained in this study, and it has been determined that plants grown in the in vitro culture method contain higher amounts of total protein (the results are of significance). It has been suggested that increases in protein content may be associated with reductase-type enzymes [30]. Total protein analysis was performed in this context due to the positive correlation between reductase-type enzymes and the synthesis, accumulation, and other non-enzymatic antioxidants of phenolic compounds. This suggests that biotechnological methods, such as plant tissue culture or in vitro cultures, are more effective than traditional cultivation methods in producing secondary metabolites or valuable bioactive substances.

It has been stated that proline is among the low molecular non-enzymatic antioxidants [45,46], and it is also known to be one of the basic defense mechanisms of plants against stress conditions [47]. Different amounts of proline accumulation have been observed in *Hypericum* species grown at different temperatures and growing conditions [48,49,50,51,52,53]. Proline accumulation has been linked to increased levels of oxidative stress [35,36,40]. Additionally, studies on different plant species grown in both natural and in vitro environments have shown that plants cultivated in vitro have a higher proline content [54]. In this study, the highest amount of proline has been determined in the treatment group grown in vitro by the tissue culture method (the results obtained are of significance), and it is in accordance with the literature data. Plants grown by the in vitro culture method may have more physiological, biochemical, and antioxidant content because they synthesize more bioactive substances to protect their physiological and biochemical processes. Therefore, by establishing continuous cultures using tissue culture methods, greater quantities of bioactive compounds can be obtained, and these valuable phytochemicals offered by nature’s pharmacy can provide more raw materials for fields such as pharmacology, medicine, and food.

Lipid peroxides, products of lipid peroxidation and free radical(s) can be produced in plant species grown in different environments and in vitro culture conditions [55]. It has been reported that the end product formation due to lipid peroxidation is MDA, and it is used as a biomarker for oxidative stress in *H. perforatum* and other *Hypericum* species [56]. The presence of phenolic compounds and other non-enzymatic antioxidants in plants is generally associated with increased levels of oxidative stress [55]. Changes in MDA levels have been observed in studies on various *Hypericum* species [50,51,57]. The studies also reported that high MDA levels indicate increased oxidative stress in in vitro plants and, consequently, compensatory activation of antioxidant systems [39,40].

Benson [55] has stated that in vitro cultures formed using different plant explants produce toxic products due to lipid peroxidation, through lipid peroxides, and free radicals.

In the present study, differences in MDA levels have been observed in *H. bilgehan-bilgilii* species grown in different growing environments (the results obtained are of significance), and, in accordance with the literature, a positive correlation was found between the group with the highest MDA level and an increase in oxidative stress. Therefore, it is thought that antioxidant defense systems, both enzymatic (such as SOD and CAT) and non-enzymatic (such as phenolic compounds and carotenoids), are activated in response to increased oxidative stress, thereby helping to counteract this adverse condition.

Plant secondary metabolites containing at least one hydroxyl group and an aromatic ring are known as phenolic compounds [58,59]. It has been noted that phenolic compounds obtained from medicinal and endemic plant species have recently attracted increasing interest due to their considerable benefits for human health [60]. The effectiveness of phenolic compounds as antimicrobial, anticancer, and antioxidant agents has been reported in studies [61]. It is believed that the number of phenolic compounds with medicinal and bioactive properties will increase in the coming years. Therefore, it is important to determine the amounts of phenolic compounds or to improve the amounts of phenolic compounds obtained from medicinal plants using biotechnological methods for use in the treatment of various diseases, such as cancer.

The amounts of TPC vary significantly in different structures of the plant (such as leaves, flowers, and stems), depending on different environmental and growing conditions and different developmental stages [34,62,63,64]. Total phenolics were analyzed from extracts obtained from different plant parts of the *H. heterophyllum* plant species under different growth conditions. In a study, it has been stated that the highest amount of TPC has been observed in seedlings obtained by the in vitro culture method [42]. In the study conducted on *H. perforatum* by Yaman, Onlu, Ahmed and Erenler [34], it has been reported that plants grown under different growing conditions have different amounts of TPC. The study indicated that higher amounts of vanillic acid were obtained in in vitro cultures, and it is known that vanillic acid is phenolic in origin. This suggests that phenolic compounds can be produced in greater quantities in in vitro cultures [52]. Numerous studies have been carried out on different *Hypericum* species obtained from different growing conditions or methods, and in these studies, it has been determined that TPC variations have been observed due to factors such as different growing conditions and different environmental conditions [15,65,66,67,68,69,70,71]. In a study conducted on *Curcuma larsenii* and *Kaempferia larsenii* plants obtained from nature and grown in vitro, it has been reported that the highest total phenolic amounts have been observed in plants grown in vitro [72,73]. It has been reported that TPC increases are also directly proportional to total antioxidant amounts or activities. In this study, the highest TPC has been determined in plants grown in the in vitro environment (the results obtained are of significance), and it has been observed to be consistent with the literature data provided above. Due to their medical attractiveness, medicinal plants cultivated using in vitro culture technologies are thought to be able to provide valuable phytochemicals, primarily phenolic compounds found in nature’s pharmacy, and biomass for pharmacological studies, as well as guarantee a continuous supply of plant material.

PAL is a highly important regulatory enzyme that mediates the production of a wide range of phytochemicals through the phenylpropanoid pathway in plants [74,75,76]. It has been reported that increased PAL activity is usually associated with phenolic compounds, resulting in high antioxidant activity [77]. In studies conducted on different *Hypericum* species, it has been stated that PAL activities increase more in in vitro cultures, and this leads to an increase in the amount of phenolic compounds [78,79,80,81,82]. This situation indicates that in vitro cultures are more advantageous in increasing bioactive substances and substances with antioxidant properties. In addition, PAL activities are also increased in in vitro cultures of different plant species, and in a study on the *Plantago ovata* plant, it has been stated that PAL enzyme activity is higher in in vitro callus cultures [83]. In this study, it has been determined that the treatment group with the highest PAL enzyme activity is plants grown in an in vitro environment, and the data of this study are consistent with the data found in the literature (the results obtained are of significance). It is known that PAL activity promotes the production of phenolic compounds via the phenylpropanoid pathway. This study confirms this finding, as the phenolic compound and PAL data are similar. It is thought that by better understanding the complex mechanisms in the phenylpropanoid pathway, more phenolic compounds can be synthesized. Future studies in this aspect will enable the large-scale production of phenolic compounds, which are offered by nature’s pharmacy and are more abundant in medicinal and endemic plants, for use in fields such as food, medicine, and pharmacology.

The interaction between enzymatic and non-enzymatic antioxidant defense systems in different plant species, from the most primitive to the highest, plays an important role in overcoming different stressors [84]. In a study conducted on *H. calycinum* species, it has been reported that SOD and CAT activities are low, but PAL enzyme activity is high in in vitro cultures of the related species. In the study, the amount of TPC has also been noted to be high in in vitro cultures [85]. In a study conducted on the *H. rumeliacum* plant species, it has been reported that enzymatic antioxidant activities (SOD and CAT) decreased in plants obtained using the tissue culture technique, and this is accompanied by an increase in non-enzymatic antioxidants [50,51]. In this study, SOD and CAT enzyme activities have been determined in the least amount in the treatment group grown under in vitro conditions, followed by an increase in the amount of non-enzymatic antioxidants. It is thought that this may be due to the complex relationship between the enzymatic antioxidant mechanisms and non-enzymatic antioxidant mechanisms pathways. It is known that many bioactive substances in *Hypericum* species are of non-enzymatic antioxidant (phenolic compounds, phenylpropanoids, carotenoids, etc.) origin. Therefore, the results obtained in this study are in line with the literature data (the results obtained are of significance). Although the enzymatic antioxidant activities of SOD and CAT were low in the in vitro lab group, the relatively high levels of non-enzymatic antioxidants, such as total phenolics, PAL, carotenoids, and DPPH, are thought to be closely related to the phenylpropanoid pathway regulation mechanism. The relevant pathway is thought to cause an increase in PAL enzyme activity, thereby triggering other non-enzymatic antioxidant defense systems associated with PAL. Studies in the literature also suggest that low SOD and CAT enzyme activities in vitro culture environments indicate the activation of other pathways. Based on the results obtained from this study and subsequent studies, valuable products from nature’s pharmacy found in medicinal plants that contain large amounts of valuable secondary metabolites, such as phenolic compounds and carotenoids, which are important for both health and pharmacology, can be produced on an industrial scale to supply raw materials to various industries.

The DPPH method is widely used to detect the antioxidant activity of extracts of different plant species regarding radical scavenging activity. It has been stated that DPPH is a stable single electron-containing radical that reacts with antioxidant substance(s) (generally, a color change from dark purple to yellow can be seen) [86]. In the DPPH radical scavenging activity analysis performed on different extracts obtained from *H. heterophyllum* and *H. perforatum* plant species grown in wild and in vitro conditions, the best DPPH radical scavenging activity has been determined in plant structures (callus) formed under in vitro conditions [34,42]. Kwiecień, et al. [87] determined the highest DPPH radical scavenging activity in plants grown under in vitro conditions in their study on *H. perforatum* plants. Numerous DPPH radical scavenging activity assays have been performed on different *Hypericum* species, and it has been stated that *Hypericum* species are a very important source of antioxidants and therefore have medicinal uses [88,89,90,91]. In a study using the plant *H. perforatum*, micropropagation of the plant has been performed in vitro, and subsequently, the DPPH activities of the in vitro culture and the donor plant have been compared. At the end of the study, it has been determined that the highest DPPH radical scavenging activity has been observed in plants grown in vitro [74]. The results obtained in the current study are consistent with this research in terms of the higher DPPH activity observed in in vitro cultures.

In this study, the best DPPH radical scavenging activity has been determined in plant extracts grown under in vitro conditions (the results obtained are of significance), and the results obtained in the study are in parallel with the literature data. It is thought that this study will shed light on the future medicinal and pharmacological studies of the *H. bilgehan-bilgilii* species with high antioxidant activity. One of the most important active compounds found in *Hypericum* species, hypericin and hyperforin, are phenolic compounds. It is thought that this encourages these plants to have greater antioxidant activity. It is thought that medicinal and endemic plants living in harsh natural conditions, such as the plant species used in our study, produce more antioxidant substances to combat stress factors and, consequently, contain higher amounts of phytochemicals. When all these characteristics are considered together, the importance of these valuable phytochemicals offered by nature’s pharmacy for human health is tremendous.

Increased amounts of carotenoids in plants have been implicated in the quenching of singlet oxygen and thus prevent oxidative damage [92]. Carotenoids have also been reported to stabilize the structure of the membrane(s) in chloroplasts by neutralizing excess energy in photosystems Ι and ΙI [93]. It has been reported that *H. rumeliacum* and *H. tetrapterum* plants grown in vitro contain high amounts of carotenoids. It has been stated that these high amounts of carotenoids protect the plant against oxidative stress [94]. The amount of carotenoids is high in *H. perforatum* species grown in vitro [95]. According to the results obtained in this study, the highest amount of carotenoids has been found in *H. bilgehan-bilgilii* plant extracts grown under in vitro conditions (the results obtained are of significance). The data obtained are similar to the results of the studies conducted in the literature. Additionally, since carotenoids are non-enzymatic antioxidants and have numerous health benefits, they are one of the most investigated components in scientific research. It is known that these valuable phytochemicals, offered by nature’s pharmacy, are found in greater quantities in medicinal plants. Due to these properties, they have potential applications in fields such as pharmacology, food science, and medicine.

Recent studies have indicated that plants may play a role in combating oxidative stress and various diseases, such as the aging process, due to their antioxidant properties [96]. Consequently, polyphenolic compounds and their derivatives, carotenoids, phenylpropanoids, and flavonoids are among the phytochemicals that receive the most attention. It has been stated that phenolic compounds, when consumed up to 1.0 g per day in a diet rich in fruits and vegetables, have inhibitory effects on mutagenesis and carcinogenesis in humans [97]. Plants belonging to the *Hypericum* genus are known to be a rich source of phenolic compounds and their derivatives. For this reason, *Hypericum* species are considered a promising source of natural antioxidants and are regarded as a valuable botanical resource offered by nature’s pharmacy for human health and various industries.

## 5. Conclusions

In this study, new information was obtained for the first time regarding the physiological, biochemical, and antioxidant properties of *H. bilgehan-bilgilii* leaf extracts obtained under different cultivation methods. Global climate change is causing more severe consequences for plant species that are endangered or endemic. By establishing in vitro cultures of plants with medicinal potential and endangered species, these plants are both protected, and their valuable bioactive components are sustainably utilized, providing natural and new raw materials for various industries. In this study, it was determined that the local endemic *H. bilgehan-bilgilii* species contains high amounts of phenolic compounds and antioxidants. When the results are evaluated together, it is found that plants obtained through plant tissue culture methods have higher amounts of physiological, biochemical, and antioxidant content compared to traditional cultivation conditions. Therefore, the physiological and antioxidant properties detected in higher amounts in the in vitro cultures of the *H. bilgehan-bilgilii* plant species will shed light on future comprehensive studies.

## Data Availability

All data represented in this work is contained within the manuscript.

## Abbreviations

The following abbreviations are used in this manuscript:

GA	Gibberellic acid
2,4-D	Dichlorophenoxy acetic acid
BAP	Benzyl amino purine
BSA	Bovine serum albumin
TP	Total protein
MDA	Malondialdehyde content
TPC	Total phenolic content
PAL	Phenylalanine ammonium lyase
SOD	Superoxide dismutase
CAT	Catalase
DPPH	2,2-Diphenyl-1-picryl hydrazyl
SA	Statistical analysis
